# Elite lean athletes at sports high schools face multiple risks for mental health concerns and are in need of psychosocial support

**DOI:** 10.1371/journal.pone.0284725

**Published:** 2023-04-21

**Authors:** Carolina Lundqvist, David P. Schary, Emelie Eklöf, Sofia Zand, Jenny Jacobsson

**Affiliations:** 1 Department of Behavioral Sciences and Learning, Linköping University, Linköping, Sweden; 2 Athletics Research Center, Linköping University, Linköping, Sweden; 3 Department of Physical Education, Sport and Human Performance, Winthrop University, Rock Hill, SC, United States of America; 4 Department of Health, Medicine and Caring Sciences, Linköping University, Linköping, Sweden; University of Valencia, SPAIN

## Abstract

The combined demands on an adolescence in an elite sports high school can negatively affect mental health (eg, stress, burnout, depression, anxiety). Late adolescence is also when elite-striving athletes typically transition from junior-to-senior level programs. In addition, adolescent elite lean sports athletes have an increased risk of suffering from abnormal eating pathology. The purpose of this study was to investigate the perceived psychosocial needs that young, elite-striving lean sports athletes in sports high schools find essential for their sports careers and transition from junior-to-senior level sports. Eight Swedish elite-striving lean sports athletes (2 men and 6 women; median age 17.0 years, range: 16–18 years) participated in this study and sports represented were athletics (n = 1), gymnastics (n = 3), and wrestling (n = 4). Semi-structured interviews were performed, and data was analyzed by thematic analysis. Results showed integration, both in the present situation and for the future, as an overarching theme perceived as essential for a successful elite sports career over time. Three additional themes were also identified: (a) Psychosocial stress (combined performance demands, diminished social life, sports-related body weight demands, taboo talking about eating disorders, injuries), (b) Protective psychosocial factors (social support and psychological safety, communication and coordination school and sports, self-care/health behaviors), and (c) support needs junior-to-senior transition (career advice and mentorship, individualized support). As a result, elite-striving, lean sports athletes attending sports high schools need additional support to excel in their academic and athletic endeavors. Sports developmental programs continue to provide inadequate support before and during the junior-to-senior level transition. Similarly, despite the continued calls for reducing the stigma of mental health, athletes in sports schools still encounter barriers from coaches and peers, making the subject taboo, particularly surrounding eating pathologies.

## Introduction

### Sports schools

The importance of protecting the wellbeing and mental health of high-performance athletes from junior-to-senior elite level is receiving attention in the scientific literature and among major sports organizations worldwide [1–3]. Sports schools provide academic services tailored to athletes’ needs of simultaneously getting education while continuing their sports careers and are intended to reduce the number of conflicting academic and athletic demands. In addition to athletic and academic support services, athletes at sports schools usually get access to higher quality training partners, role models, and more intense training and competition routines [4]. In Sweden, there are Swedish national sports high schools governed by the Swedish National Sports Confederation and approved by the National Board of Education [www.rf.se] where elite-striving athletes aged 16 to 19 are selected through national recruitment. Athletes at these schools complete a regular national high school program combined with an elite sports education. Because the sports high schools are located in certain places in Sweden, many young athletes move from home to live in a boarding school during their studies.

Research shows that the combined demands of elite sports and school can negatively affect mental health (eg, stress, burnout, depression, anxiety), skew perceptions of performance expectations, and increase the risk of injuries and illness [4–6]. Findings are, however, not consistent and some studies indicate relatively low levels of stress among athletes [7], as well as better general health and well-being [8, 9]. Regardless, almost all researchers recognize the importance of a holistic approach that allows young athletes to develop competencies, skills, and identities that extend beyond sports into other domains of life [4, 10]. Positive talent development to pursue wellbeing, in addition to personal and athletic development, are characterized by a supportive, psychological safe and empowering environment with knowledgeable coaches and staff, as well as communication and good relationship between various stakeholders [11].

### Junior-to-senior transition

It is known that the junior-to-senor transition is a challenging phase during an elite sports career [12]. This period involves psychosocial stressors, for example, higher levels of competition and performance expectations, along with changing social relationships with coaches or teammates [12–14]. Adjustment difficulties are common during this transition phase, but intraindividual variation is expected due to personal dispositions, availability of resources, and environmental supports [12].

Studies show that the peak onset age for several common mental disorders is in the mid-teens to the mid-20s [15]. For most elite athletes, the junior-to-senior transition also occurs during this same time, late adolescence or early adulthood [14], which is a vulnerable developmental phase. As a result, there are unique, non-sports specific psychosocial stressors that occur during this time. These can include changing relationships with parents, moving away from home, greater responsibilities for taking care of oneself, and greater academic or vocational demands [16]. There is also increasing commercialization and professionalization within contemporary youth sports, which has led to stakeholders and media putting more demands and obligations on younger athletes [12, 17]. Since the developmental path from novice to an elite level can vary considerably among athletes and mean age for the transition to senior sports also varies across sports [12], these stressors are not uniformly experienced, and may fluctuate between being present or absent during different career phases.

### Disordered eating, eating disorders and lean sports

During adolescence, male and female athletes are more susceptible to developing abnormal eating pathologies compared to non-athletes [18–21]. Prevalence studies vary in their results [22, 23], but athletes participating in lean sports have an increased risk of suffering from abnormal eating pathology [21, 24–26]. Lean sports include endurance (eg, long distance running, triathlons), aesthetic (eg, gymnastics, diving), and weight-dependent sports where low body weight may improve performance (eg, wrestling, lightweight rowing) [26]. In addition to these sports-related risk-factors, other interrelated etiological factors for abnormal eating pathologies include biological (genetics), psychological/personality (eg, perfectionism, self-esteem, body-dissatisfaction, low tolerance level for negative affect), developmental (eg, puberty, history of abuse or bullying, stressful life-events), and socio-cultural factors (eg, thin body ideal) [27, 28].

Eating behavior can vary on a spectrum from optimized nutrition to disordered eating (DE) to clinical eating disorders (ED) [24]. DE is characterized by various pathogenic thoughts about food and weight-control behaviors like restrictive eating, diets, and compensatory behaviors to burn or get rid of calories. Thus, DE are abnormal eating behaviors that do not fully meet the criteria for ED but still have negative psychological, physical and performance effects [24]. Moreover, DE is a predisposing factor for athletes to develop ED and early identification and intervention are beneficial for positive treatment results and prognosis [29, 30].

ED are specified by criteria in the Diagnostic and Statistical Manual of Mental Disorders-5 Text Revision (DSM-5 TR) [31]. They are often severe, protracted, and difficult-to-treat psychiatric conditions in need of specialist care [24, 32]. Among the ED found in DSM-5 TR, athletes are most reported to suffer from anorexia nervosa and bulimia nervosa [22]. Anorexia nervosa is a severe condition linked to an increased mortality rate [28] and characterized by a severely restricted caloric consumption compared to caloric requirements, as well as an intense fear of weight gain and denial of seriousness despite a significant low body weight [31]. Core symptoms of anorexia nervosa are body image disturbances with a self-evaluation highly reliant on body weight and denial of the seriousness of one’s low body weight. Bulimia nervosa is also characterized by a self-evaluation highly influenced by one’s body weight and shape, but behaviors include binge eating episodes with a lack of control and overeating followed by inappropriate compensatory behaviors to prevent weight gain (eg, excessive exercise, self-induced vomiting, misuse of laxatives or other medication to get rid of the food eaten when binging) [31].

### Study purpose

Elite-striving adolescence athletes in sports high schools and competing in lean sports face multiple challenges (ie, general challenges during adolescence, being an athlete with dual performance demands, moving towards junior-to-senior transition, meeting increased risk-factors for DE/ED). This implies a multiple vulnerability for mental health concerns. Unfortunately, there are still limited amounts of research specifically targeting elite and elite-striving lean sports athletes enrolled in sports schools, particularly during the junior-to-senior transition phase. Consequently, little is known about this specific sub-population of athletes. The purpose of this study was therefore to investigate the perceived psychosocial needs that young elite-striving lean sports athletes in a sports school find essential for their elite sports careers and transition from junior-to-senior sports.

## Materials and methods

This study adopted a qualitative research design to investigate the subjective experiences of young elite-striving lean sports athletes in Swedish sports high schools. The study was approved by the Swedish Ethical Review Authority (Dnr 2020–06259) before data collection. Results are reported according to the Consolidated Criteria for Reporting Qualitative Research (COREQ) [33].

### Participants

Participants were recruited by contacting representatives of the national sports high schools, who under the auspices of the Swedish National Sports Federation, conducts education for various sports including lean sports. Representatives were provided an informational letter about the study and ethics via email, and in turn, shared this information with their athletes. A total of eight Swedish elite-striving lean sports athletes (2 men and 6 women; median age 17.0 years, range: 16–18 years) participated. Sports represented in this sample were athletics (n = 1), gymnastics (n = 3), and wrestling (n = 4). All athletes were studying at Swedish national sports high schools, were ranked in the national top levels for their age, and one had participated on the senior national team. Based on the Swann and colleagues’ [34] definition of an elite athlete, the participants were semi-elite, taking part in talent development programs (ie, elite-striving) in three separate schools located in different parts of Sweden. Demographic characteristics of all participants are presented in Table 1. Participants who agreed to participate in the study provided their written informed consent prior data collection and that the interviews were recorded.

**Table 1:** Participant demographic.

Participant #	Gender	Sports	Sports level	Participated senior national team
1	Female	Wrestling	National junior elite	No
2	Male	Wrestling	National junior elite	No
3	Male	Wrestling	National junior elite	No
4	Female	Wrestling	National junior elite	No
5	Female	Gymnastics	National junior elite	Yes
6	Female	Gymnastics	National junior elite	No
7	Female	Gymnastics	National junior elite	No
8	Female	Track-and-field	National junior elite	No

### Interview guide and data collection

Data was collected during spring 2021 and interviews were performed and recorded using Zoom. All interviews followed a semi-structured interview guide developed by three of the authors (CL, EE, SZ). The first author is an Associate Professor in both psychology and sports sciences, as well as a clinical psychotherapist licensed by the by the Swedish National Board of Health and Welfare. The first author also has extensive experience of applied work with sports- and clinical psychology in elite sports settings. The third and fourth author (EE, SZ) are clinical psychologists with an interest in sports psychology.

The main themes of the interview guide covered (a) demographic information, sports experiences, and goal setting within the sports (eg, “Can you tell us about your sporting background?”, “Can you tell us about your short- and long-term goals with doing your sport?”), (b) understanding and experiences of mental health (e.g., “What comes to mind when you hear the word mental health? Mental health in sports?”, “Do you feel that your sports participation affects your mental health in any way [positively and/or negatively])?”, and (c) experienced support and support needs in the present situation, as well as related to the junior-to-senior transition (eg, “What do you think about psychological/sports psychology support as part of a sports investment?”, “Have you received or been offered any kind of psychological intervention or training aimed at preparing you for the future as an elite athlete at the senior level?”). The full interview guide is available from the first author.

Participants were asked to reflect on and discuss the interview topics, follow-up questions and probes were used to encourage the respondents to develop and deepen their answers so that informative interview data could be obtained. The third and fourth author (EE, SZ) performed all interviews together. The interviews varied in length from 32 minutes to 1 hour and 33 minutes. All interviews were transcribed verbatim by the third and fourth author (EE, SZ) and the accuracy of the transcripts were thereafter verified by the first author (CL). All audio files were permanently deleted after transcription.

### Data analysis and rigour

Data was analysed inductively, underpinned by a pragmatic epistemology [35], using thematic analysis which is appropriate for relatively homogenous samples [36, 37]. The aim of thematic analysis is to uncover qualitative themes characterized by “stories about particular patterns of shared meaning across the dataset” [37, p. 592]. The data-analysis followed the steps suggested by Braun and Clarke [36] and searched to identify latent content in terms of underlying assumptions and ideas in the interviews. The third and fourth author (EE, SZ) transcribed the data and read and re-read the transcripts to carefully engage with data. They also collaboratively identified codes, which was then followed by generating initial themes. The first author (CL) was subsequently reading the transcripts and reflected on codes and initial themes generated by the third and fourth author.

During the ongoing data-analysis, all three authors repeatedly discussed the relevance of generated themes to gradually deepen the latent content and to decide if candidate themes could be separated, collapsed, or rearranged. During these discussions, the interview transcripts were also scrutinised by the authors to ensure that no relevant data was overlooked and to make sure that themes were coherent with patterns of meaning found in the data. These discussions continued until consensus was reached and themes meaningfully could be arranged in a thematic map. The themes were then named.

Following Tracy’s [38] recommendations, rigour was central throughout the entire process of planning the study, collecting data, data-analysis, and presenting the results. Participants from the target sample were invited because they were anticipated to provide insightful perspectives in relation to the research question. The authors also searched throughout the data-collection and analysis phase to ensure that enough data was collected, and carefully analysed in appropriate depth, to enable rigorous support for the study findings. A sample size of 6–10 interviewees is recommended for small research projects [39], and in this study, data-collection stopped after eight interviews based on the decision that theoretical saturation occurred related to the objective of this study. During the reporting of the results, the authors worked to carefully present the findings to enable a significant knowledge contribution. During this phase, the second (DS) and fifth author (JJ) individually reviewed and critically evaluated the featured themes and their organisation to ensure a meaningful coherence in the presentation of the results.

## Results

**Fig 1:**
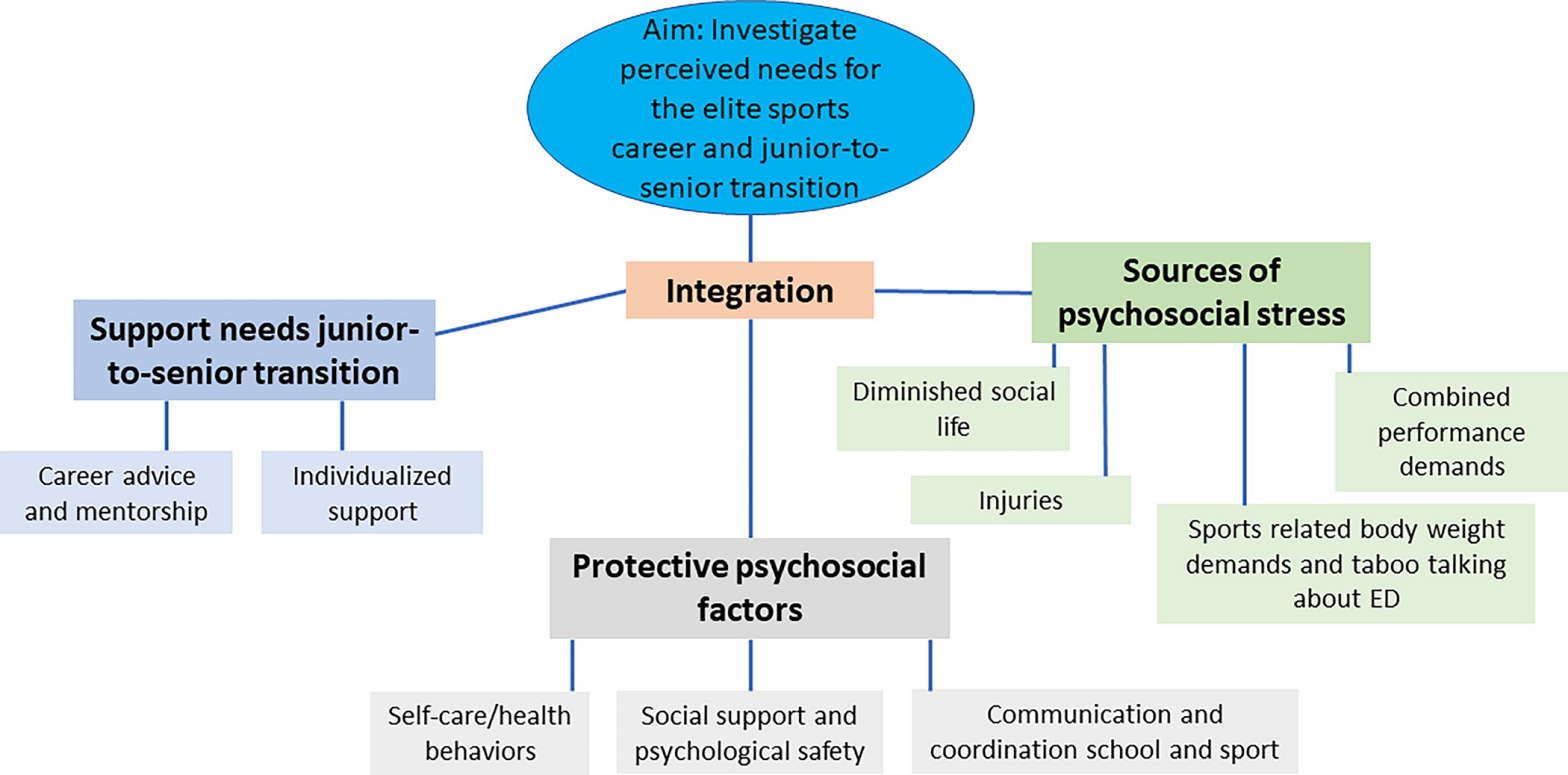
Thematic map of the results.

The thematic map of the results is shown in Fig 1.

### Integration

An overarching theme identified during the data analysis was a sense of integration, both in the present situation and for the future, which was seen as essential for a successful elite sports career over time. The theme of integration encapsulates when pieces of life and sports fit well together with necessary support available, resulting in life and sports satisfaction, a sense of development, and well-functioning in the environment with a general positive outlook on life and future opportunities.

“I think that if you want to keep your head in place for a longer period of time, you may need to get some help sometimes or learn how to work with yourself” (# 4).

The theme of integration also related to the perceived relationship between coping with various career and life phases, to put effort in sustaining your mental health, seek support when needed, and formed a basis for continued development and performance. The importance of integration was visible in the athletes’ definitions of mental health when they were discussing the construct and how it was expressed in their everyday life.

“…that you feel good, you feel confident with yourself, and you are positive about life. A bit like that” (# 1)

“… how you feel on the inside when you train, with yourself, how you feel and everything you feel. I kind of think about what’s going on in your head and how comfortable you are in the situation and how you enjoy yourself” (# 8)

### Sources of psychosocial stress

Psychosocial stress was generated as a major theme among the participants with four sources identified: (a) Combined performance demands, (b) Diminished social life, (c) Sports-related body weight demands and ED being a taboo topic, and (d) Injuries.

### Combined performance demands

Multiple performance demands in sports and school combined to increase the participants’ level of perceived stress. A reoccurring stressor was perceived time constraints. A significant amount of time was spent in practice and competition, limiting their ability to keep up with their academic work. The participants, however, still felt pressure to perform well in school. This tension could result in the athletes feeling inadequate in both domains. In addition, it was common among the interviewed athletes to experience performance anxiety related to competitions.

“You need to manage your studies at the same time as you need to manage to train seven times a week and then perhaps also compete. It is demanding and there is not enough time” (# 2).

Being enrolled at a sports high school increased the demands on the athletes, especially at a boarding school because of the added responsibility of managing daily tasks (eg, cooking, laundry, cleaning). The format of sports boarding schools, where most athletes live and train together, also heightened interpersonal comparison related to, for example, athletic development, academic achievement, and health habits like amount of sleep and nutrition. These interpersonal comparisons appeared as a prominent and underlying cause of perceived stress among the participants, and as a potential risk-factor for developing a negative view of one’s development and achievements.

“You feel the pressure from home because you’ve come here to show that you’ve become something. Then maybe you haven’t developed as fast as the others and it’s tough. Because there’s a lot you can compare with others and maybe you just notice other peoples’ successes but not your own” (# 1).

Participating in their sports could also be experienced as a double-edged sword. While the performance requirements were perceived as high, sports were also a positive outlet to improve their mental health, a place where the athletes felt they got to release other sources of life stress and where the physical activity itself was perceived as facilitative for their wellbeing.

“One positive thing is that when I practice my sport, I become much more relaxed and can let go of everything and just focus on training” (# 4).

### Diminished social life

The athletes also stated that they struggled with a healthy life-sports-school balance, increasing the risk of a narrowed identity centered on performing in sports and school, which could increase feelings of loneliness or boredom. A diminished social life was recurringly expressed in the interviews. Normal and restorative social activities that typical adolescence participate in, like meeting friends outside sports or school activities, were often absent.

“It can be very difficult to achieve this balance and the social part is not enough. It’s easy to think only of the training and studies and performance” (# 8).

A lack of social life could also result in the participants being vulnerable to declined wellbeing if the group climate at school or in sports was perceived as unhealthy.

“If you don’t have a good environment. When I wasn’t feeling well, the group was bad as well. There were two people who loved when people argued and always tried to create some irritation in people and said things that were not true” (# 4).

Moreover, the predominant performance focus could result in the athletes neglecting social relationships that serve as a protective factor to their overall mental health. Not stopping and reflecting on their own wellbeing was also common, posing an increased risk of not detecting stress-related signals at an early stage that can contribute to poorer wellbeing over time.

“It’s mainly that you end up in your own bubble and it’s that bubble that I think is dangerous” (# 1).

### Sports-related body weight demands and taboo talking about ED

A predominant source of stress among the athletes were the sports-related body weight demands and the perceived pressure or hype around their body weight. The perceived weight focus could be communicated directly or indirectly from coaches, stressing the importance of weight, body, and diet.

“Some coaches say it outright, but from some it slips out, like, you need to think about eating more vegetables or something like that. Some just say you’re too big and you need to lose weight” (# 7).

Thoughts about body weight and shape could be exaggerated if the athlete had a successful role model who was very thin. This increased the participants’ own beliefs that being thin was necessary to be successful. The focus on body weight and shape could also increase due to the body being exposed in tight clothing or when competing in weight classes.

“I think a lot about the weight classes, that you must hold back. It easily ends up with thoughts like I’m getting too big now I must hold back, also when it’s not even the competition period. I am just feeling it anyway. You stand on the scale and, shit, now I’ve gained half a kilo, then you get a little stressed” (# 4).

Stress around body weight and eating behaviors could build when seeing other athletes struggling with DE or ED, increasing fear of being affected yourself.

“In my former club, there were several who had quit because of anorexia. In my sport there are quite many who are affected by anorexia and eating disorders and such, then you get a bit scared that you might accidentally get into it yourself” (# 6).

An additional area of concern, which increased the athletes’ stress and anxiety around the issue was whether it was perceived to be taboo talking about ED. Despite being prevalent, ED were seen as a sensitive topic in which coaches’ knowledge and skills to respond to the issue were lacking, subsequently posing a risk that the topic was avoided instead of being addressed.

“It’s quite taboo, but if someone has an eating disorder, you know about it. But no one would ever bring it up and talk about it. It’s so sensitive, because maybe the coach has suffered from it [herself or himself] and does not want to bring it up. Because the coach isn’t very good at talking about that sort of things. It is very taboo” (# 7).

### Injuries

Being injured was a great source of stress that negatively impacted the athletes’ wellbeing. Throughout the interviews, the participants revealed that a high prevalence of injuries, particularly overuse injuries, were an accepted and normalized part of their sports. A predominant stressor while being injured were the athletes’ perception of their own athletic stagnation while seeing other athletes continue to develop and perform well.

“Right now, I think it [my wellbeing] is somewhat negative because I’m injured. Then I am thinking that [without the injury] I could have been at the same level and trained and competed like everyone else” (# 5).

Injuries could increase stress levels, but stress could also be perceived as a factor contributing to injuries, becoming a barrier to recovery. Thus, injuries and stress become a vicious circle, negatively affecting each other and subsequently draining the athletes’ wellbeing over time.

“Past year I was injured the complete year, almost the whole season. My shoulder was dislocated, and I think it had to do with my stress. That it was part of the reason why it happened and why it was difficult to recover” (# 1).

### Protective psychosocial factors

A third theme that was generated from the interviews were protective psychosocial factors for the athletes’ mental health, these included: (a) Social support and psychological safety, (b) Communication and coordination school and sports, and (c) Self-care/health behaviors.

### Social support and psychological safety

Participants reported that social support, which often extended beyond those involved in their sports, was essential to their wellbeing. The most prominent sources of social support were immediate family (ie, parents and siblings), coaches, teammates, and friends. A strong social support system provided the participants with a sense of psychological safety, reducing the stress and giving them the space to develop as a person and athlete.

“Both coaches and parents, they are the main ones /…/ I know they are there, and I know they support me. They do everything to make me feel good and just to feel that safety is important” (# 3).

Emotional support from their social network was of utmost importance to the athletes. This included having a supportive adult in their social network (eg, coach, parent, teacher). The participants felt accepted when they had an adult they trusted and could discuss major personal matters like mental health concerns, as well as daily stresses experienced in their dual roles as student and athlete.

“I would say our coach because I have managed to open a bit to him. I can tell him that today I’m having a really bad day. Then I get to choose for myself how much I want to participate in and can handle. You are an athlete and even though you should feel good, it’s okay to feel bad and it’s okay to admit it” (# 4).

This emotional support also strengthened the participants’ sense of self-value unrelated to sports performance and protected their self-esteem when confronted with body weight or body shape demands in the lean sports environment.

“It’s my boyfriend, he assures me that I’m fine even though I’m not super skinny” (# 7).

A recurring topic emerging in the interviews was the perceived need to have regular access to some professional psychology support provider (eg, mental health counselor, sports psychologist, psychologist) to which the participants could express their feelings, as well as receive additional emotional support and guidance on how to cope with various performance demands.

“You may not always feel that you need it, although I think the truth is the opposite. That it’s actually more important than you think, unfortunately, or yes it’s good but… I absolutely think it’s important” (# 2).

Participants also expressed the need for informational social support, getting guidance and advice related to sports, school, and general life issues. The informational support providers the athletes turned to varied greatly (eg, coaches, teachers, parents, friends, mental health counselor, sports psychologist), suggesting that their choice was reliant on the perceived match between the immediate issue and the person in their network who was available or seen as the best position to provide guidance about the issue. Moreover, the availability of tangible support to combine sports and everyday chores made the life-situation easier to manage.

“My parents have helped me shop a few times so that I can go do the shopping once a month. So that I don’t have to buy all the pasta and all the rice and stuff” (# 7).

### Communication and coordination between school and sports

The unique combination of school and sports provided by sports schools were perceived as beneficial if there was scheduling flexibility when the load was temporarily higher in one of the domains (eg, several competitions in a row, exams in school).

“The teachers at school can adapt and understand if you have had a stressful time, if it was a competition or if you are preparing for a competition. Or if you hold back on food in the last week before weigh-in, they can still understand and adapt their lessons, according to what I can do and what I need” (# 4).

Good communication between different coaches, as well as between individuals in the school and sports settings, were stressed by the participants as a significant factor in reducing stress and getting everyone working towards the same goal.

“I think it is important with communication between my coach in athletics and the sports school coaches, I think there is a gap there that means [pause] it could be better. It could be an improved overall situation and improved planning” (# 8).

An autonomy supportive climate also appeared as an important factor that the athletes perceived could help them stay motivated and maintain their wellbeing over time. A recurring theme in the interviews was a desire for an increased opportunity to create balance between training, competition, and recovery. This included being able to take a break from sports to get time for other opportunities.

“To be able to perform, I want to have the possibility to go away and have a holiday. So I can let go of everything and then come back with full energy. Just let a few things go and come back and continue at full speed. If I think it’s a bit too much and a bit hard, then to do something you think is fun instead. It’s more difficult to go away and do something now” (# 5).

### Self-care/health behaviors

It was apparent across all the interviews that self-care and facilitative health behaviors (eg, sleep, nutrition) helped protect the participants’ wellbeing over time and increased their development as an athlete. The athletes used several techniques they had discovered themselves or had learned from a teacher, coach, sports psychologist, or mental health counselor. Planning and time management skills were expressed as necessary to be able to cope with the multiple performance requirements and to maintain functional daily routines.

“Structuring my days… When it was as hardest, when I basically had difficult to stay awake… We have someone at school who helps with structuring the school, whom I will start meeting soon. So we’re going to start structuring school and sports and things like that” (# 1).

Other skills/techniques athletes adopted to protect their mental health related to stress reduction was, for example, the use of breathing exercises, and focusing on one thing at a time instead of multi-tasking. In addition, participants mentioned the use of distraction when feeling stressed or when being disappointed with the performance.

### Support needs junior-to-senior transition

The final theme generated from the analyses was support needed to prepare for the junior-to-senior transition. The two perceived needs for a successful transition from junior-to-senor level were (a) Career advice and mentorship, and (b) Individualized support.

### Career advice and mentorship

Participants reported that preparations for the junior-to-senor transition were rare and reliant on individual efforts by coaches who chose to discuss the transition phase with their athletes.

“So there has been a lot of talk with my coach about how I should do it. That now you’re a senior and you have demands on yourself because now you’re competing… It’s your first year, you don’t have to prove who you are now” (# 5).

Overall, the participants received limited formal education or targeted support on how to prepare for the junior-to-senior transition. This made them feel abandoned, left alone to find their own way.

“I think that more support like that is needed and that you talk more about the future and this transition. How to continue with your sport because I have no knowledge of that. I don’t know how to continue and how to think or something” (# 8).

Most participants wished they had received career advice and mentorship from someone experienced, like older athletes or coaches, who could explain the process, what to expect, and how to successfully move into senior sports. Moreover, it was viewed as beneficial to get the opportunity to start preparing for the transition process in an earlier phase of development.

“To be able to talk to someone experienced and gain insight into how it can be before you are there yourself, I think it would be best. So that you know what you’re getting yourself into. Otherwise, it’s like a big black wall that you must walk straight through, it becomes quite difficult. /…/ so that you always have a guide and don’t walk around in the dark and fumble. It is very important to have someone who can show you the way” (# 2).

### Individualized support

A need for continued, and more individualized, support after graduating from the sports school and moving towards senior sports was apparent in most interviews. Some athletes were concerned they would lose their important emotional support network of coaches, teachers, teammates, and other support personnel.

The request for higher levels of individual support was also a consequence the support received at the sports school. The support was perceived as scattered, athletes expressed their desire for someone who could be responsible to oversee and advise them on major decisions.

“It becomes so scattered and how I should do and how I should think. There is a lot going on in this head. Okay, am I right or wrong, but I still make conscious choices. /…/ I can do a lot and I kind of know what I’m doing. But I haven’t been doing training or is an educated trainer. Therefore, I can sometimes feel quite alone with my thoughts” (# 8).

Overall, the participants anticipated that the pressure and demands would increase at the senior level and that individualization of support (eg, physical, mental, nutrition) would be needed. The exact combination of desired support depended on the athlete’s goals for future development.

“Access to support of various kinds, whether it’s physical, psychological… you might need to work a lot with mental training. You work a lot with physical training, but you may not think about the mental training, and it is just as important” (# 1).

## Discussion

The present study investigated perceived psychosocial needs that young elite lean sports athletes in a sports school find essential for their elite sports careers and transition from junior-to-senior sports. Results revealed an interplay between mental health, performance in school, and performance in sports, each of which influence each other in a reciprocal manner. Another notable finding in our study was the participants’ need to obtain a sustainable lifestyle that balanced sports, school, and other life responsibilities when living in a boarding school with no parents around. This was necessary to maintain their wellbeing and develop as a person and athlete. Consistent with findings in previous studies [4], results in this study showed that the athletes enrolled in sports high schools can become narrowly focused on performance requirements, resulting in a diminished social life, increased levels of stress, and undermined recovery. Performance demands in dual domains (ie, school and sports) required time-management skills, coping strategies, and the use of self-care behaviors to protect mental health and to manage the demands of school and sports. In line with a growing number of sports studies [3], perceived psychological safety, often in combination with emotional, informational, and tangible social support from coaches, family and friends, was also mentioned by the participants as essential to protect their mental health and development throughout their sports high school experience.

As has also been revealed in previous research on positive talent development environments [11, 40], good communication and a well-functioning infrastructure between school and sports, with an opportunity to individually adjust the workload and get recovery when needed, was greatly desired among the participants but seldom obtained. In addition, training and living together with other athletes in a boarding school increased the risk of maladaptive comparisons with peers regarding school, sports, and health-related factors. These comparisons further imposed a risk of accumulated perceived stress and dissatisfaction with one’s own development and performance. Altogether, the current study supported several of the findings in previous studies [4] suggesting that athletes at sports high schools are likely to face multiple stressors that potentially can threaten mental health and risk diminishing long-term opportunities for optimal athletic development.

Moreover, challenges associated with the junior-to-senior transition are well-known in research, showing that it is common for athletes in a range of sports and nationalities to drop-out or stay static in development, and that this period is known to require both personal and environmental resources for athletes to successfully adjust to the senior level competition [41–44]. Nonetheless, athletes in the present study still described that they had received scare formal preparation for the transition, but wanted career advice, mentorship, and individual support based on their unique needs for development. Sports federations, sports high schools, coaches, and other support staff around athletes in this phase should pay careful attention to the athletes’ needs. This includes creating environments that emphasize long-term and holistic development, as well as an emphasis on the importance of mental health. Sports high schools are in an excellent position to provide specific programming that can meet the current needs of their athletes and prepare them for transitioning to senior level competition.

In addition to studying adolescence sports high school athletes confronting junior-to-senior transition, this study also explored the perspectives of elite-striving lean sports athletes. Researchers have previously found DE and ED to be prevalent in lean sports [26], which calls for attention to minimize risk factors and increase preventive measures for these late adolescence athletes being in a vulnerable age. Over the last several years, significant efforts from researchers and leading international sports organizations have tried to reduce the stigma around mental health issues, emphasizing the need to consider physical and mental health as equally important [3, 45]. This was, however, not the reality for participants in this study. Although DE and ED were central to participants’ everyday life, it was expressed as taboo to talk about in sports environments. Coaches were perceived by athletes to lack adequate knowledge or skills to manage DE and ED, and could express insensitive comments about the athletes’ weight or body shape, which is a risk-factor for DE or ED for vulnerable athletes [46]. Consequently, athletes in this study expressed hesitation to address any issue regarding eating concerns with their coaches.

The findings around DE and ED are troublesome because previous studies have shown that mental health stigma in elite sports environments reduces help-seeking behaviors among athletes [47]. ED are known to be most treatment-responsive in the early stages of development. When entrenched, it will require more intensive treatment, is more intractable, or even unremitting particularly when related to severe weight loss, binge eating, and vomiting [29]. Thus, early detection and a short history is a favorable prognostic factor for treatment. Unfortunately, early detection is undermined by perceived stigmas or lack of suitably trained support staff that athletes can go to for help.

The results found in this study support claims by Haslam et al. [30] that education and training resources are still needed to support coaches, support staff, and sports organizations to decreases risks with athletes developing DE and ED. It is important to recognize DE and ED as a health and safety issue [24], and to stress the importance of preventive measures combined with evidence-based education regarding proper weight and nutrition to reduce the risk of DE and ED in elite sports. Coaches and support staff should also be made aware of that lean sports athletes are especially vulnerable to DE and ED because of the intense preoccupation often present in these sports on energy, nutrient intake, and strategies to manipulate body composition or the necessity to maintain a lean body shape for performance purposes [23]. It is also known that nutrition knowledge does not necessarily transfer into behavior. As a result, ED can be a one of many factors that prevent athletes from eating adequately, despite being knowledgeable of nutrition [48, 49].

DE and ED are conditions affecting all aspects of health (mental, social, physical) and is related to increased incidence of injuries and reduced performance over time [24, 50]. An additional source of concern found in the present study was that injuries were expressed as being part of youth sports, thus a phenomenon that risks becoming normalized. Perceived ill-health related to injuries have been reported to be high in sports high school settings [51, 52] and stress is one factor that has been associated with injuries amongst dance students [53]. Furthermore, research suggests that injuries related to overuse may be partly a consequence of inadequate self-perception among elite athletes, supporting the need for psychological interventions in injury prevention research [54].

The overall findings in this study indicate the importance of a proper support systems infrastructure around high school lean sports athletes with appropriately educated support staff that can meet the athletes’ multiple needs related to school and sports. A multidisciplinary team should include professionals that can provide knowledge that extends beyond general nutrition information. They should be experienced in prevention, to identify and treat eating pathology among athletes. It is essential to remember that ED can develop into severe psychiatric conditions and require licensed health professionals with adequate competence in core symptoms and evidence-based treatments.

The findings also stress the importance of proper knowledge transfer of research to significant persons close to the athletes. This includes providing coaches and other support staff with greater knowledge of risk-factors for eating pathologies, especially in lean sports. Moreover, coaches should develop their coaching effectiveness in terms of interpersonal knowledge with proper communication and relationship with athletes of different ages and contexts, as well as interpersonal knowledge, and being able to reflect on own work to nurture a healthy relationship with their athletes [55]. Sports schools have the additional responsibility of having proper support personnel and programs, as well as logistical systems that reduce unnecessary burdens on their students. This will allow them to develop as whole people, not just athletes or students. To improve the health and performance of future elite athletes, all stakeholders in elite sports must escalate their efforts to provide positive, holistic developmental environments for athletes.

Although this study yielded several interesting findings that support and build on previous research, it is not without limitations. Importantly, the results are from a small sample of elite-striving athletes enrolled in Swedish sports high schools. As a result, their experiences may differ from other athletes in different countries and regions of the world. Researchers and practitioners should interpret the findings within the appropriate cultural framework. Similarly, the data was gathered and analyzed using qualitative research methods. This allows for a detailed account of each participant’s experience but limited the breadth of experiences. Quantitative research can capture general trends among a larger sampled size of athletes, which may help researchers and practitioners address areas of need. Future research should examine the experiences of athletes in sports high schools in other countries and diverse populations using a variety of methodologies, including mix-methods and longitudinal designs.

The data was collected in the spring 2021 during the height of the COVID-19 pandemic. While Sweden had limited social distancing restrictions compared to other areas of the world, this unique time limited travel and competition. Coupled with the stresses involved with the pandemic, the participants’ experiences during the data collection may have altered their perceptions of needs, particularly regarding their mental health. Post-pandemic, future research should examine the experiences and perceived needs of elite-striving athletes enrolled in sports high schools to see if there are differences.

The exploratory and inductive nature of this study fits well with thematic analysis because the goal was to uncover the perceived needs of the athletes. Utilizing different frameworks, however, may help uncover relationships between themes. For example, adopting an ecological perspective [40, 56] may help to discover conditions both in micro (eg, peer relationships) and macro (eg, school culture) environments that can act as resources or barriers for athletic and personal development, as well as mental health. Future research should adopt different theoretical frameworks to better understand the challenges facing elite and elite-striving athletes.

In conclusion, the results of this study suggest that elite-striving, lean sports athletes attending sports high schools need additional support to excel in their academic and athletic endeavors. Knowledge translation is the science aiming to close the gap between research and practice [57]. However, there are recognized challenges when it comes to the dissemination and adoption of research results into practice and barriers for this shortcoming needs to be identified in the specific context applied [57]. The findings in our study expose that national developmental programs continue to provide inadequate support despite having the knowledge and directives regarding its importance before and during the junior-to-senior level transition. Similarly, despite the continued calls for reducing the stigma surrounding mental health, athletes still encounter barriers from coaches and peers, making the subject taboo. For lean sports athletes, the mental health stigma makes it difficult to counter and address DE and ED. This is particularly concerning for elite-striving athletes because of their age and developmental level, eating pathologies can have a lasting negative effect on their health and performance. Unfortunately, this shows that empirical knowledge itself does not automatically ensure application by coaches or sports schools, where it can make a difference for athletes.
